# Integrating clinical pharmacists in care management for secondary stroke prevention clinical trials: a scoping review

**DOI:** 10.3389/fneur.2025.1666409

**Published:** 2025-09-17

**Authors:** James Thurston, Hanlin Li, Ian M. Kronish, Imama A. Naqvi

**Affiliations:** ^1^Department of Pharmacy, NewYork-Presbyterian Hospital, New York, NY, United States; ^2^Department of Medicine, Columbia University Irving Medical Center, NewYork-Presbyterian Hospital, New York, NY, United States; ^3^Department of Neurology, Columbia University Irving Medical Center, NewYork-Presbyterian Hospital, New York, NY, United States

**Keywords:** clinical pharmacist, stroke, multidisciplinary, interdisciplinary, telehealth

## Abstract

**Introduction:**

Clinical pharmacist (CP) integration within interprofessional healthcare team models may effectively provide secondary stroke prevention care and address healthcare disparities.

**Methods:**

This scoping review includes randomized controlled trials (RCTs) that evaluated the effect of interventions inclusive of team-based care by CPs on patient-oriented and health outcomes after stroke or transient ischemic attack (TIA). Search databases included MEDLINE/PubMed, EMBASE, and CINAHL, and ClinicalTrials.gov and the International Standard Randomised Controlled Trial Number (ISRCTN) trial registries. We describe the level of interaction between interprofessional team members, use of telehealth services, population diversity, and intervention effects on study outcomes.

**Results:**

Of 132 RCTs, 14 met inclusion criteria and incorporated CPs in the intervention. These studies were conducted globally and included outcome measures such as medication adherence, morbidity and mortality, and vascular risk factor goal attainment. Twelve trials included multidisciplinary models, while two included interdisciplinary models, and none incorporated transdisciplinary models. Telehealth was leveraged in 8 of 14 trials. One study reported on healthcare disparities associated with poor risk factor control. Positive intervention effects were notable for goal attainment (4 of 10 trials).

**Discussion:**

Published RCTs examining CP impact within secondary stroke prevention teams with limited data suggests that interventions inclusive of CPs delivering medication education, reconciliation, and titration may improve vascular risk factor control, medication adherence, and patient-oriented outcomes. We highlight the need for future secondary stroke prevention clinical trials to provide more insight into CP integration, promote diversity in study populations and clinician roles, and incorporate telehealth to enhance healthcare access.

## Introduction

Each year in the U.S., about 795,000 strokes occur, including 185,000 recurrent events (1). Most are preventable, with 90.5% of the global stroke burden linked to modifiable risk factors like hypertension, diabetes, and dyslipidemia (2). Recent guidelines support tailored risk factor management and multidisciplinary, team-based care to enhance secondary stroke prevention (3).

Racial and ethnic minorities face a disproportionate burden of vascular risk factors and higher stroke recurrence rates due to healthcare inequities. These populations often encounter barriers to care such as access to medications, language challenges, mistrust of healthcare, low health literacy, and systemic racism (4–6). Team-based care may address these disparities through coordinated, patient-centered services (7, 8).

**Figure 1:**
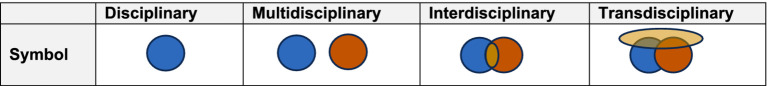
Types of team-based models. Disciplinary defined as independent pharmacist services without collaboration; multidisciplinary defined as multiple disciplines working in coordinated, yet separated services; interdisciplinary defined as multiple disciplines working together to provide care simultaneously; transdisciplinary defined as disciplines working together with less defined healthcare roles and services that transcend these traditional discipline roles (12).

Traditionally, neurologists have led post-stroke care, but an aging population (9) and neurologist shortages (10, 11) highlight the need for interprofessional co-management. Within collaborative team-based models, team dynamics differ by disciplinary interaction (Figure 1). “Multidisciplinary” team works in parallel, while “interdisciplinary” signifies integrated services and “transdisciplinary” describes roles sharing across disciplines (12).

Clinical pharmacists (CPs) are highly accessible medication experts who can support complex medication education and management at transitions of care (13–15). Within ambulatory post-stroke care, CPs can titrate medications, monitor adherence, and order labs to optimize risk factors. While all pharmacists hold advanced degrees (e.g., PharmD) and licensure, CPs can provide more advanced ambulatory care services, often administered through in-person or telehealth visits. Further, under Collaborative Practice agreements (CPAs), they can independently prescribe and manage medication therapy (16).

There is limited research that explores CP integration into secondary stroke prevention, particularly regarding health equity and telehealth (17). This review evaluates RCTs involving CP-inclusive care teams within secondary stroke prevention and examines the diversity of studied populations (18). Findings may inform future models to improve adherence, prevent recurrence, and reduce disparities in post-stroke care.

## Methods

**Figure 2:**
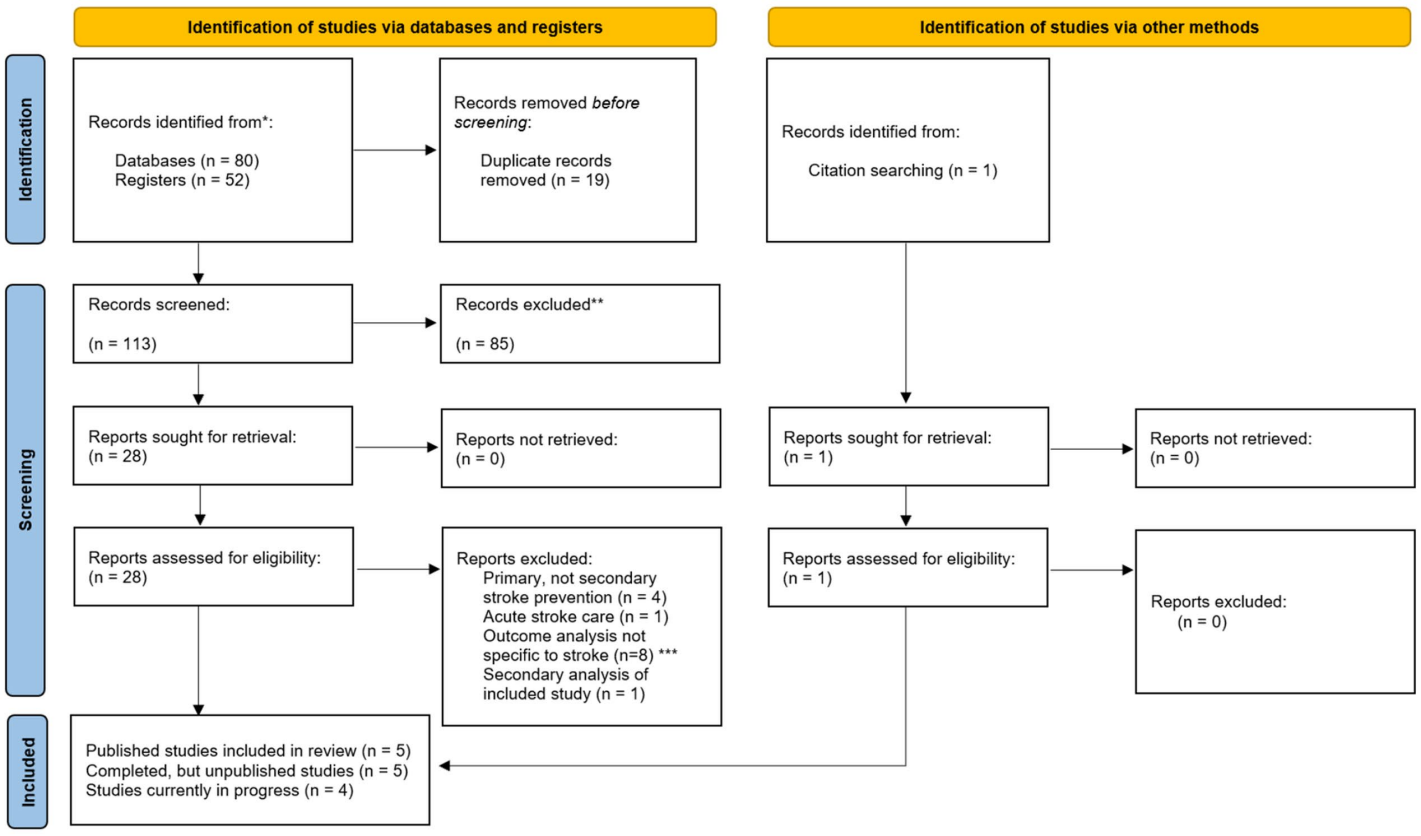
PRISMA diagram (Preferred Reporting Items for Systematic Reviews and Meta-Analyses) including searches of databases, registers and other sources. *Databases included Ovid MEDLINE/PubMed, EMBASE, and CINAHL. Trial registers included ClinicalTrials.gov and International Standard Randomized Controlled Trial Number (ISRCTN). This included all studies submitted to the trial registers and/or published from database inception until 12 March 2024. **Reasons for record exclusion include studies that examined primary stroke prevention, did not include outpatient visits in the intervention, or did not examine stroke-specific outcomes. ***Outcome analysis not specific to stroke included any composite cardiovascular outcomes that included conditions outside of stroke (e.g., Sudden cardiac arrest).

We conducted a structured scoping review to summarize the range and characteristics of research evaluating interventions inclusive of CPs to improve secondary prevention outcomes in patients with stroke and transient ischemic attack (TIA). We chose a scoping review for this purpose instead of a systematic review to capture trial designs, interventions, and outcomes of all posted studies to guide future research and practice priorities. Our scoping review followed reporting guidelines of Preferred Reporting Items for Systematic reviews and Meta-Analyses Extension for Scoping Reviews (PRISMA-ScR) (Figure 2; Supplementary material) (19).

### Search strategy

We searched Ovid MEDLINE/PubMed, EMBASE, CINAHL, ClinicalTrials.gov, and ISRCTN using the query: “((stroke OR transient ischemic attack) AND (pharmacist OR pharmacists)),” filtered for randomized controlled trials (RCTs). Searches included from the trial registries and/or published from database inception until 12 March 2024. Additional studies were identified by manually searching bibliographies from included articles.

### Selection of studies

We included RCTs enrolling adults (≥18 years) with stroke or TIA that tested secondary prevention interventions involving CPs in ambulatory settings. Studies were excluded if they focused on primary prevention, did not specify stroke-related outcomes, lacked ambulatory care components, or were non-randomized, observational, or non-English. Economic and process evaluations were excluded from the systematic search but are referenced in the discussion. An additional RCT abstract was identified from a recent systematic that focused on pharmacist roles in both primary and secondary prevention through 2021 but excluded ongoing trials (17, 20).

Three authors (J.T., H.L., I.A.N.) independently screened titles, abstracts, and full texts, reaching consensus at each stage. Data extraction was performed by one author (J.T.) using a standardized form and reviewed by two others (H.L., I.A.N.) for accuracy. These included information about the study site, study methods, patient population, interdisciplinary model, mode of delivery for patient visits, CP interventions, CP scope of practice (prescribing authority. no prescribing authority), interventional phase of care (assessed as time since stroke event), outcome measures, and study results. Study authors also extracted information on the timing and duration of intervention of any qualifying stroke event.

Consistent with a scoping review, evaluation of the methodological quality for each study was not conducted with the intention to include all available evidence. A narrative account was gathered by intervention type and outcomes with a focus on pharmacist engagement in team-based care models.

## Results

### Results of the search

Of 132 unique randomized controlled trials identified, 14 RCTs met inclusion criteria for this review – 13 through search strategies and one through manual selection (17, 20). Eight studies were excluded with outcomes analyses not specific to stroke, such as composite cardiovascular health outcomes that included, for example, cardiac arrest in addition to stroke.

### Included studies

Of the 14 RCTs included, five are published with results (8, 21–24), four have been posted but are still pending results (25–28), one study has only been published as an abstract (20), and four studies are posted in trial databases, but still in progress (29–32). We included a secondary analysis of one of these original studies within our review, but did not consider this as a separate RCT for inclusion (23, 33).

**Figure 3:**
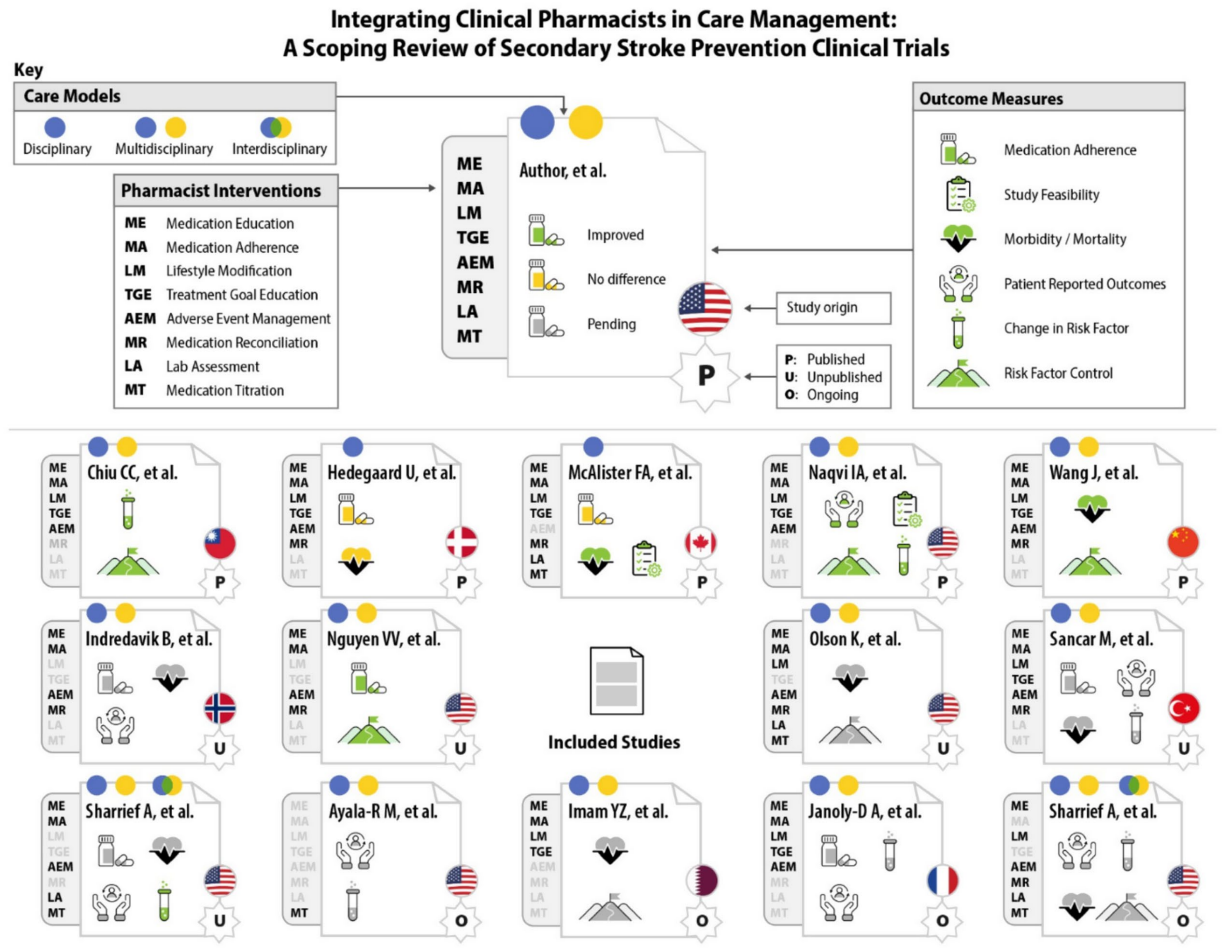
Graphic representation of randomized clinical trials engaging clinical pharmacist interventions and outcome measures for secondary stroke prevention.

All 14 studies included at least one site considered as an urban setting, while three studies (25, 30, 32) included sites in suburban settings (34). While numerous studies reported demographic data, only one study targeted these disparities (8). This study identified patient factors among their study population that are associated with poor blood pressure (BP) control, such as Black and Hispanic race/ethnicity, lower socioeconomic status, and low health literacy (8, 35, 36). Race and ethnicity reporting from all RCTs is reported in Table 1. A visual summary of all results is depicted in Figure 3.

**Table 1:** Secondary stroke prevention RCTs demographics report.

Article citation	Study location	Urban/suburban/rural Setting^#^	Reported study demographics	Health insurance use	Education level	Primary language
Published clinical trials with results						
Chiu CC, et al. (21)	Guishan District, Taoyuan City, Taiwan	Urban	50% female Mean age 65.3 years	Not reported	46% illiterate	Not reported
Hedegaard U, et al. (22)	Odense, Denmark	Urban	61% female Mean age 66 years	Not reported	Not reported	Not reported
McAlister FA, et al. CMAJ. 2014 (23) McAlister FA, et al. Am Heart J. 2014 (33)	Edmonton, Alberta, Canada	Urban	42% female Mean age 67.6 years	Not reported	Not reported	Not reported
Naqvi IA, et al. (8, 37)	Washington Heights, New York City, NY, USA	Urban	36% female Mean age 64.3 years 44% Hispanic 32% Black 20% White	26% Medicare 28% Medicaid 30% private insurance 8% uninsured	54% less than or equal to a high school education	English and Spanish
Wang J, et al. (24)	Shijiazhuang, Hebei Province, China	Urban	49% female Mean age 60.7 years	46.4% insured 53.6% uninsured	55% ≤ 9 years of education	Not reported
Complete clinical trials pending published data						
Indredavik B, et al. (25)	Kristiansund, Levanger, Molde, Namsos, Trondheim, and Ålesund, Norway	Urban/suburban	Not reported	Not reported	Not reported	Not reported
Nguyen, VV, et al. [abstract]. (20)	Los Angeles, USA	Urban	Not reported	Not reported	Not reported	Not reported
Olson K, et al. (26)	Aurora, Colorado, USA	Urban	Not reported	Not reported	Not reported	Not reported
Sancar M, et al. (27)	Istanbul, Turkey	Urban	Not reported	Not reported	Not reported	Not reported
Sharrief A, et al. (STOP-Stroke) ID#: NCT03923790 (28)	Houston, Texas, USA	Urban	52% female Mean age 54.5 years 45% Black 31% White 24% Hispanic or Latino	68.7% uninsured	Not reported	Not reported
Ongoing clinical trials						
Ayala-Rivera M, et al. (32)	Downey, Sylmar, Torrance, and Los Angeles California, USA	Urban/suburban	In progress	In progress	In progress	In progress Only included patients who were able to speak English or Spanish
Imam YZ, et al. (29)	Doha, Qatar	Urban	In progress	In progress	In progress	In progress
Janoly-Dumenil A, et al. (30)	Bron, Cébazat, Echirolles, Paris, Saint Genis Laval, and Saint-Etienne, France	Urban/suburban	In progress	In progress	In progress	In progress
Sharrief A, et al. (VIRTUAL). ID#: NCT05264298 (31)	Houston, Texas, USA	Urban	In progress	In progress	In progress	In progress

^#^Urban defined as >50,000 inhabitants with 1,000 people per square mile, suburban defined as 2,500–50,000 inhabitants, and rural defined as <2,500 inhabitants (34)^.^

### Intervention timing and duration

Most (11 of 14) study designs incorporated a 6-month (6 of 14 studies) (20–24, 31) or 12-month (5 of 14 studies) (25, 27, 29, 30, 32) intervention period. Clinical pharmacist interventions occurred within three months of a stroke event in two studies (8, 25), within six months post-stroke in four studies (22, 24, 28, 31), and within 12 months post-stroke in two studies (27, 30). Conversely, one study only looked at patients who had sustained a chronic stroke event at least 12 months prior to any intervention (21). Three studies (23, 29, 32) included interventions for patients less than or greater than 12 months post-stroke and two studies (20, 26) did not specify duration.

### Clinical pharmacist roles

Of the 13 studies that described the CP roles, the most common CP contribution included medication education/counseling (13 of 13 studies), adherence assessment and education/counseling (12 of 13 studies) (8, 20–30), lifestyle education/counseling (10 of 13 studies) (8, 21–24, 26, 27, 29–31), and identification of medication-related adverse effects (10 of 13 studies) (8, 21, 22, 24–28, 30, 31). The least common were lab assessment (4 of 13 studies) (23, 26, 28, 31) and medication titration (5 of 13 studies) (23, 26, 28, 31, 32). Four of these studies (23, 26, 31, 32) confirmed that CPs had expanded practice scope, allowing them to independently make these medication titration decisions for patients. One other study that included medication titration as an intervention did not specify this (28). Clinical pharmacist intervention details are summarized in Table 2.

**Table 2:** Clinical pharmacist interventions in secondary stroke prevention trials.

Article citation	Clinical pharmacist visit model	Visit frequency	Did clinical pharmacist have scope to adjust medications	Types of clinical pharmacist interventions	Which phase of care for clinical pharmacist interventions
Published clinical trials with results					
Chiu CC, et al. (21)	In-person outpatient	Monthly one-hour CP education program for up to 6 months	No	Disease state/treatment goal education Medication education Identification of med-related adverse effects Adherence assessment/education Lifestyle education/modification	>12 months post-stroke
Hedegaard U, et al. (22)	In-person at discharge In-person outpatient Telephone call visits	One in-person visit at hospital, followed by phone visits at 1 week, 2 months, and 6 months	No	Disease state/treatment goal education Medication education Identification of med-related adverse effects Medication reconciliation Adherence assessment/education Lifestyle education/modification	<3 months post-stroke 3–6 months post-stroke
McAlister FA, et al. CMAJ. 2014 (23) McAlister FA, et al. Am Heart J. 2014 (33)	In-person outpatient	Followed at monthly visits for up to 6 months	Yes	Disease state/treatment goal education Medication education Medication reconciliation Adherence assessment/education Lab assessment Medication titration Lifestyle education/modification	<3 months post-stroke 3–6 months post-stroke 6–12 months post-stroke >12 months post-stroke
Naqvi IA, et al. (8, 37)	Video visits	Two CP visits, at 4 and 8 weeks after hospital discharge NP visit at 1–2 weeks and physician visits at 6 and 12 weeks	No	Disease state/treatment goal education Medication education Identification of med-related adverse effects Adherence assessment/education Lifestyle education/modification	<3 months post-stroke
Wang J, et al. (24)	In-person at discharge In-person outpatient Telephone call visits Video visits	One discharge consultation, followed by one monthly visit for 6 months	No	Disease state/treatment goal education Medication education Identification of med-related adverse effects Medication reconciliation Adherence assessment/education Lifestyle education/modification	<3 months post-stroke 3–6 months post-stroke
Complete clinical trials pending reported data					
Indredavik B, et al. (25)	In-person at discharge Telephone call visits	Interview at discharge followed by visits after 1 week, 1 month, 2 months, and 3 months post-discharge	No	Medication education Identification of med-related adverse effects Medication reconciliation Adherence assessment/education	<3 months post-stroke
Nguyen, VV, et al. [abstract] (20).	Telephone call visits	One visit at 3 months and one visit at 6 months from time of randomization	No	Disease state/treatment goal education Medication education Adherence assessment/education	Unclear/not documented
Olson K, et al. (26)	Unclear/not documented	Unclear visit frequency	Yes	Medication education Identification of med-related adverse effects Medication reconciliation Adherence assessment/education Lab assessment Medication titration Lifestyle education/modification	Unclear/not documented
Sancar M, et al. (27)	In-person at discharge In-person outpatient	Discharge visit followed by outpatient visits every 3 months	No	Disease state/treatment goal education Medication education Identification of med-related adverse effects Medication reconciliation Adherence assessment/education Lifestyle education/modification	<3 months post-stroke 3–6 months post-stroke 6–12 months post-stroke
Sharrief A, et al. (STOP-Stroke) ID#: NCT03923790 (28)	In-person at discharge Telephone call visits Video visits	Discharge visit followed by visits after 1 week, 1 month, 3 months, and 5 months post-enrollment More frequent phone visits may be required based on BP measurements	Unclear	Medication education Identification of med-related adverse effects Adherence assessment/education Lab assessment Medication titration	<3 months post-stroke 3–6 months post-stroke
Ongoing clinical trials					
Ayala-Rivera M, et al. (32)	Unclear/not documented	Monthly health coach calls, unclear how often pharmacist visits will be held for medication titration/management	Yes	Medication titration	<3 months post-stroke 3–6 months post-stroke 6–12 months post-stroke >12 months post-stroke
Imam YZ, et al. (29)	Unclear/not documented	Visits scheduled initially every month, then every three months, for one year	No	Disease state/treatment goal education Medication education Adherence assessment/education Lifestyle education/modification	<3 months post-stroke 3–6 months post-stroke 6–12 months post-stroke >12 months post-stroke
Janoly-Dumenil A, et al. (30)	In-person at discharge In-person outpatient Telephone call visits	Discharge visit followed by telephone visits after 3 months, 6 months, and 9 months post-discharge, then a final in-person interview at 12 months	No	Disease state/treatment goal education Medication education Identification of med-related adverse effects Adherence assessment/education Lifestyle education/modification	<3 months post-stroke 3–6 months post-stroke 6–12 months post-stroke
Sharrief A, et al. (VIRTUAL). ID#: NCT05264298 (31)	Telephone call visits *(Control Group)* Video visits *(Intervention Group)*	Intervention Group: Scheduled video appointments at 1–2 weeks, 1 month, 3 months, and 5 months with team PLUS remote telemonitoring of BP with BP med adjustments biweekly as needed by CPs Control Group: Scheduled independent telephone visits with CP once monthly for first 6 months	Intervention Group: Yes Control Group: No	Medication education Identification of med-related adverse effects Medication reconciliation Lab assessment Medication titration Lifestyle education/modification	<3 months post-stroke 3–6 months post-stroke

### Types of team-based models

Most studies included multidisciplinary or interdisciplinary components, while no studies had transdisciplinary components. In two studies, CPs saw patients independently, without close coordination with any other healthcare professionals (22, 23). Twelve studies instead included a multidisciplinary model, allowing CPs to work with other disciplines in coordinated, but separate services (8, 20, 21, 24–32). Two of these 12 studies also utilized interdisciplinary pharmacist care, in which CPs worked together with other healthcare professionals to provide care during the same visit (28, 31). In both cases, the control group was designated as the multidisciplinary component, while the interventional group was the interdisciplinary component.

### Mode of delivery

Modes of care delivery varied across studies, and each study often included multiple types of delivery in their methods. These forms of care delivery included telephone visits (7 of 14 studies) (20, 22, 24, 25, 28, 30, 31), in-person ambulatory visits (6 of 14 studies) (21–24, 27, 30), in-person visits at discharge (6 of 14 studies) (22, 24, 25, 27, 28, 30), and video visits (4 of 14 studies) (8, 24, 28, 31). The mode of delivery could not be assessed based on available information for two of the included ongoing studies (29, 32).

### Intensity of intervention visits

For six out of 14 included studies, ambulatory CP follow-up visits were scheduled more frequently at the start of study periods (i.e., weekly or bi-weekly), followed by less frequent visits (i.e., monthly or quarterly) (22, 25, 28–31). In some cases, patients were only seen once monthly (4 of 14 studies) (8, 21, 23, 24) or once every three months (2 of 14 studies) (20, 27) from the start of the study period. Two of the studies that have not reported results also did not report the frequency of CP visits (29, 32). “Usual Care” differed significantly across studies, and follow-up schema that was specifically noted in the studies are listed in Table 3.

**Table 3:** Clinical pharmacists integration in secondary stroke prevention: detail of trials, measures and results.

Article citation	Study methods	Description of CP care integration	Outcomes measures	Results
Published clinical trials with results				
Chiu CC, et al. (21)	Trial Design: Prospective RCT Time Frame: 6-months Control: 80 patients seen by PCP Intervention: 80 patients seen by PCP and CP	Independent CP and PCP visits	Primary: Differences in BP, A1c, BG, TG, TC, and LDL before and after study Secondary: Proportion of patients with pre-defined adequate management/improvement in these values	Primary: Improved BP, lipid, and glycemic values by the end of the study in the intervention group. Only glycemic values improved in the control group. Secondary BP: 43 to 43% in control group vs. 31 to 65% in CP group (*p*=<0.001) Lipid: 26 to 26% in control group vs. 13 to 40% in CP group (*p*=0.01) Glycemic: 36 to 45% in control group vs. 21 to 35% in CP group (*p*=0.75)
Hedegaard U, et al. (22)	Trial Design: Prospective RCT Time Frame:6-months Control: 101 patients seen by PCP/NPs Intervention: 102 patients seen by CPs	Experimental group: Independent CP visits Control Group: Either PCP follow-up or nurse-run clinic	Primary: Overall adherence to thrombo-preventative regimen based on medication possession ratio (MPR) Secondary: Adherence to individual medications within thrombo-preventative regimen Medication persistence Combined endpoint of death, MI, or hemorrhagic or ischemic stroke	Primary: At 12 months, median MPRs were 0.95 in the intervention group vs. 0.91 in the control group—non-significant Secondary: No significant differences were found for adherence or persistence measures No significant differences were found for composite clinical endpoint
McAlister FA, et al. CMAJ. 2014 (23, 33)	Trial Design: Prospective RCT Time Frame: 6-months Control: 136 patients seen by nurse w/PCP coordination Intervention: 143 patients seen by CP alone Secondary analysis: 136 control group patients and 139 experimental group patients	Independent CP visits, who faxed updates to PCP Control group included visits with RN who would screen patient and fax information/feedback to PCP Neurologist delegated care to PCP, who delegated care to nurse in RN group	Primary: Proportion of patients who attained BP/lipid control at 6 months Secondary: Mortality, self-reported medication adherence, BMI, smoking status Secondary Analysis: 10-year risk of any vascular event using the Framingham Risk Score (FRS) and Cardiovascular Disease Life Expectancy model (CDLEM)	Primary: Improved BP and lipid control in CP group at 6 months – 43.4% in CP group met both SBP and LDL targets vs. 30.9% in nurse-led group (12.5% absolute difference; NNT=8, *p*=0.03) Secondary: No appreciable differences Secondary Analysis: FRS 10-year risk: At 6 months: median 4.8% for the CP group vs. 5.1% for the RN group (*p*= 0.44) At 12 months: median 6.4% vs. 5.5% (*p* = 0.83) CDLEM 10-year risk: At 6 months: median 10.0% for the CP group vs. 12.5% for the RN group (*p* = 0.37) At 12 months: median 8.4% vs. 13.1% (*p* = 0.20)
Naqvi IA, et al. (8, 37)	Trial Design: Parallel 2-armed prospective feasibility pilot RCT Time Frame: 3-months Control: 25 patients seen by stroke physician under “usual care” Intervention:25 intervention patients seen via telehealth by multidisciplinary team (including a CP)	Independent CP visits as part of multidisciplinary approach Experimental Group: NP visit at 2 weeks, CP visit at 4 and 8 weeks, physician visit at 6 and 12 weeks Control group: Only saw NP at 2 weeks and PCP at 6 and 12 weeks	Feasibility outcomes included recruitment, randomization, clinic adherence, and retention Clinical outcomes included change in systolic BP Patient-Reported Outcomes included Patient-Reported Outcomes Measurement Information System Managing Medications and Treatment (PROMIS-MMT), Patient Activation Measure (PAM), Neuro-QOL (Quality of Life in Neurological Disorders) Cognitive Function, Neuro-QOL Depression, and Patient Health Questionnaire-9 (PHQ-9)	Feasibility: At 3 months, adherence (91% vs. 75%, *p*=0.14) and retention (84% vs. 64%, *p*= 0.11) were higher in the intervention group Clinical Outcomes: Home SBP declined by 16±19 mmHg from baseline in the intervention group and increased by 3±24 mmHg in the control group (p=0.01) Patient-Reported Outcomes: Improved self-efficacy of medical management in all, and depressive symptoms in the intervention group
Wang J, et al. (24)	Trial Design: Prospective parallel RCT Time Frame: 6-months Control: 82 patients seen by PCP “(usual care”) Intervention: 84 patients seen by CPs in addition to PCPs (“usual care”)	Both groups: “Usual Care”: 1-month and 6-month post-discharge visit with physicians Investigational group: Independent CP visits plus “usual care”	Primary: Achievement of secondary prevention markers (BP < 140/90 mmHg, LDL-C <70 mg/dL, and HbA1c ≤ 7%) Achievement of medication adherence via Medication Adherence Report Scale (MARS-5) Secondary: Any event leading to hospitalization readmission	Primary: Improved risk factor control in CP group versus control group for A1c goal (88% vs. 53%, *p* = 0.038) and LDL-C goal (67% vs. 49%, p = 0.02). No significant difference in BP control Improved medication adherence in CP group for anti-hypertensive drugs (93% vs. 79%, *p* = 0.031), anti-diabetic drugs (92% vs. 70%, *p* = 0.02), and lipid-lowering drugs (77% vs. 61%, *p* = 0.022) Secondary: Fewer re-admissions in CP group vs. control group (7% vs. 18%, p = 0.03)
Complete clinical trials pending reported data				
Indredavik B, et al. (25)	*Completed 06/2016* Trial Design: Prospective parallel assignment RCT Time Frame: 1 year Control: “standard of care” alone Intervention: CP counseling visits plus “standard of care”	Intervention Group: Scheduled visits with hospital-based CP Unclear how information will be relayed to other health professionals	Primary: Self-reporting of adherence at 3 months Secondary: Self-reporting of adherence at 12 months, persistence (using prescription fill data), patient satisfaction, incidence of CV events and death, degree of disability or dependence in daily activities	*Not posted or reported*
Nguyen, VV, et al. [abstract] (20).	*Completed date unknown* Trial Design: Prospective RCT Time Frame: 6-months 30 total patients Control:” usual care” Intervention: CP intervention	Independent CP telephone calls CPs communicated with PCPs/stroke care provider to relay recommendations	Medication adherence based on pharmacy refill history Achievement of stroke prevention goals (BP, BG, LDL-C goals)	*Published as abstract (with results)* Medication adherence: More likely to be fully adherent in CP intervention group vs. usual care at 6 months (56% vs. 36%) Adherence to antithrombotic therapy specifically increased in intervention group at 6 months (100% vs. 88%) Achievement of stroke prevention goals: Greater goal achievement in CP intervention group vs. usual care at 6 months: BP goal (73% vs. 57%) LDL-C goal (75% vs. 50%) BG control (75% vs. 50%) This goal achievement continued or improved by 1 year mark within intervention group
Olson K, et al. (26)	*Completed 11/2018* Trial Design: Prospective parallel-assignment RCT Time Frame: 3 years Control: standard of care through PCP Intervention: CP visits alone	Intervention Group: CPs will independently conduct visits Patients requiring more in-depth dietary counseling can be referred to dieticians, or other appropriate resources PCPs will be informed of all medication initiations or dosage adjustments	Primary: Proportion of patients who attain LDL-C and BP goals Secondary: Incidence of major cardiovascular events, hospitalizations and/or death over duration of study period Tertiary: Efficacy and safety of intervention compared to usual care	*Not posted or reported*
Sancar M, et al. (27)	*Completed 4/2023* Trial Design: Prospective parallel-assignment RCT Time Frame: 1 year Control: “Standard of care” through PCP alone Intervention: CP visits plus “standard of care” through PCP	Intervention Group: CPs will independently conduct visits in coordination with stroke neurologists (on the same day) every 3 months	Primary: BP, A1c, LDL, TG, BMI, and medication adherence (Morisky-Green-Levine adherence scale) Secondary: Change in QOL Change in NIHSS Incidence of stroke recurrence and drug-related problems	*Not posted or reported*
Sharrief A, et al. (STOP-Stroke) ID#: NCT03923790 (28)	*Completed 10/2021* Trial Design: Prospective parallel-assignment RCT Time Frame:5 months Control: 41 control patients with “usual care” alone Intervention: 42 patients with “usual care” and seen at multi-disciplinary follow-up visits	Both Groups: Independent CP visit at discharge Nurse navigator call within 72 h post-discharge to assure that they have received their meds and follow-up appointments Intervention Only: Patient receives BP monitor 7-day post-discharge f/u video visit attended by MD or NP, social worker, and CP NP and CP review BP data and adjust meds SW assesses need for resources NP and CP review BP via online portal every 2 weeks until the average BP is <130/80 mmHg, then review monthly Uncontrolled BP prompts call from CP to discuss adherence and med titration Subsequent f/u visits occur 1 month, 3 months, and 5 months after enrollment	Primary: Differences in ambulatory daytime SBP Secondary: Differences in daytime DBP, nighttime BP, BMI, incidence of recurrent vascular events, and acute healthcare utilization Medication adherence (via Morisky Medication Adherence Scale), caregiver burden (via Zarit Caregiver Burden Questionnaire) Self-efficacy for taking medication as prescribed (via Medication Adherence Self-Efficacy Scale)	*Partially reported (**clinicaltrials.gov**) 11/2022* Large number of patients in both groups without outcome measures collected – incomplete results No statistical analysis
Ongoing clinical trials				
Ayala-Rivera M, et al. (32)	*Estimated completion 2026* Trial Design: Prospective parallel-assignment RCT Time Frame: 12 months Control: “Usual care” alone Intervention: Multidisciplinary visits with health coach and CP	Intervention Group: Patients receive a home BP monitor, have monthly phone calls from a health coach, and medication initiation and titration by a clinical pharmacist Control Group: “Usual care”	Primary: Change in SBP Secondary: “Life’s Essential 8” survey for cardiovascular health BMI, total cholesterol, and HbA1c California Health Interview Survey for diet, Behavioral Risk Factor Surveillance System Survey Questionnaire for physical activities, and PATH wave 1 survey for smoking,	*Reported as “In progress”*
Imam YZ, et al. (29)	*No recent updates: estimated completion 2019* Trial Design: Prospective RCT Time Frame: 12 months Control: “Standard of care” by neurologist alone Intervention: CP and stroke care NP visits plus “standard of care” by stroke neurologist	Intervention Group: Follow-up visits with CP or stroke care-trained NP who will coordinate care with stroke neurologist	*Per Published Study Design (PMID: 32664066):* Primary: Mean difference in BP and LDL Secondary: Incidence of stroke, MI, or death Carotid plaque progression as measured by 3D Carotid Doppler imaging studies	*No reported or available results*
Janoly-Dumenil A, et al. (30)	*No recent updates: estimated completion 06/2020* Trial Design: Prospective parallel-assignment RCT Time Frame: 12 months Control: “Standard of care” through PCP alone Intervention: CP visits plus “standard of care” through PCP	Intervention Group: Scheduled visits at discharge and outpatient with hospital-based CP Information from visits will be shared with PCPs and community CPs Control Group: Pharmacist will meet with patients at discharge for medication review and meet with patients at 12 months for adverse effect identification	Primary: Composite measure of medication adherence using refill data and self-reported questionnaire Secondary: Incidence of readmission and CV events Incidence of drug-related adverse effects Analysis of pharmacy refills Satisfaction of patients, providers and community pharmacists Intervention profit estimation Measure of glycemic and lipid tests	*No reported or available results*
Sharrief A, et al. (VIRTUAL). ID#: NCT05264298 (31)	*Estimated completion 2025* Trial Design: Prospective RCT Time Frame: 6 months Control: “Standard of care” PCP visits and monthly follow-up with CP Intervention: Telehealth interdisciplinary visit	Intervention Group: Scheduled follow-up visits with an interdisciplinary team member (Stroke provider, social worker, CP) Control Group: Follow-up with stroke provider within 2 weeks of discharge. Monthly BP follow-up with CP for 6 months. Follow-up with social worker following discharge	Primary: Percentage of patients with controlled BP (<125/75 mmHg) Secondary: Proportion of uninsured patients who obtain insurance Proportion of patients with controlled BP Composite incidence of recurrent vascular events Incidence of acute healthcare utilization Tertiary: Proportion of patients who quit or attempt to quit smoking; Ambulatory SBP and DBP at daytime and nighttime; PHQ-9 depressive symptoms	*Reported as “In progress”*

BG, blood glucose; BP, blood pressure; CP, clinical pharmacist; DBP, diastolic blood pressure; LDL; low-density lipoprotein; NP, nurse practitioner; PCP, primary care provider; RCT, randomized controlled trial; RN, registered nurse; SBP, systolic blood pressure; TC, total cholesterol; TG, triglycerides.

### Outcome measures

Study outcome measures were categorized by the study authors into patient-oriented outcomes, feasibility of service implementation, and clinical efficacy outcomes. Of the seven trials that reported results, six had less than 10% of study participants withdraw prior to the final follow-up visit (8, 20–24). One of these did not publish results and did not have outcomes data for greater than 30% of participants in both groups (28). An overview of the methodologies and outcomes are reported in Table 3.

### Outcome results from completed trials

Complete outcome results were available in the five of the included trials (8, 21–24), as well as the one included abstract (20). Another included RCT reported some results within the clinical trial database, but these were not statistically analyzed and interpretations could not be made due to a high patient drop-out rate (28).

Of the four studies with medication adherence results, two studies showed approximately 15 to 30% greater improvement in medication adherence rates within the CP-inclusive intervention group compared with the non-CP control group as measured by medication fill-data (20) or adherence questionnaire (24). Only one of the six trials with available results assessed other patient-oriented outcomes, such as patient satisfaction, and QOL measures, both of which improved in the intervention (CP) group (37). This study also assessed feasibility, and demonstrated that both patient adherence to study visits and patient retention were significantly higher in the multidisciplinary intervention group (91% vs. 75 and 84% vs. 64%, respectively) (8).

One study reported a composite clinical endpoint of death, myocardial infarction (MI), or hemorrhagic or ischemic stroke, which showed no significant difference between groups (22). Similarly, one study that only assessed patient mortality as an endpoint saw no appreciable difference between groups (23). One study that only assessed re-admissions as a clinical endpoint, however, showed that a lower percentage of patients within the intervention group had a re-admission within a 6-month period than the control group (7.1% vs. 18.3%, respectively) (24). Lastly, a secondary analysis of one of the studies that assessed models of future vascular event risk and life expectancy showed a non-significant difference between groups (33).

Among the five studies that reported results for patients meeting combined goals of vascular risk factor control including BP, glycemic, or lipid-lowering goals, all studies showed at least one area of significant benefit within the intervention group, without any worsened outcomes for any of these goals (8, 20, 21, 23, 24).

Of those studies that specifically assessed BP control, the percentage of patients with controlled BP by the end of the study ranged from 16% (20) to 22% (21) higher in the intervention group than the control group. Within the one study that focused on addressing healthcare racial/ethnic disparities, attainment of BP goals was specifically reported for Black and Hispanic patients, both of which had higher goal attainment in the intervention group than the control group (100% vs. 29 and 62% vs. 17%, respectively) (8, 35, 36). Another study did not demonstrate a significant difference between race/ethnicity groups, but did determine that the CP-inclusive intervention group goal attainment was nominally higher (89.3% vs. 76.8%) than the non-CP control group (24). One study looked at the combined attainment of BP and lipid control goals, which saw 43.4% attainment in the intervention group vs. 30.9% in the control group (23). This study did not examine differences between race/ethnicity.

Three studies reported lipid-lowering goals and glycemic control independent of other achieved goals, and all showed improvement in the intervention group, ranging from approximately 14–25 percentage points higher in lipid goal attainment versus the control group by the end of the studies (20, 21, 24). For attainment of glycemic goals, these were 10–35% higher in the intervention group versus the control group by the end of the studies (20, 21, 24).

In terms of absolute value change of patients’ BP measurements, the one study that examined healthcare disparities showed that intervention group patients, regardless of race/ethnicity, had average systolic blood pressure (SBP) measurements that were 13 mmHg lower than the control group by the end of the study (8). Another study reported the absolute change in BP, low-density lipoprotein (LDL) and fasting blood glucose (FBG), all of which were significantly lower by the end of the study in the intervention group, but only significantly lower for FBG in the control group (21).

## Discussion

**Figure 4:**
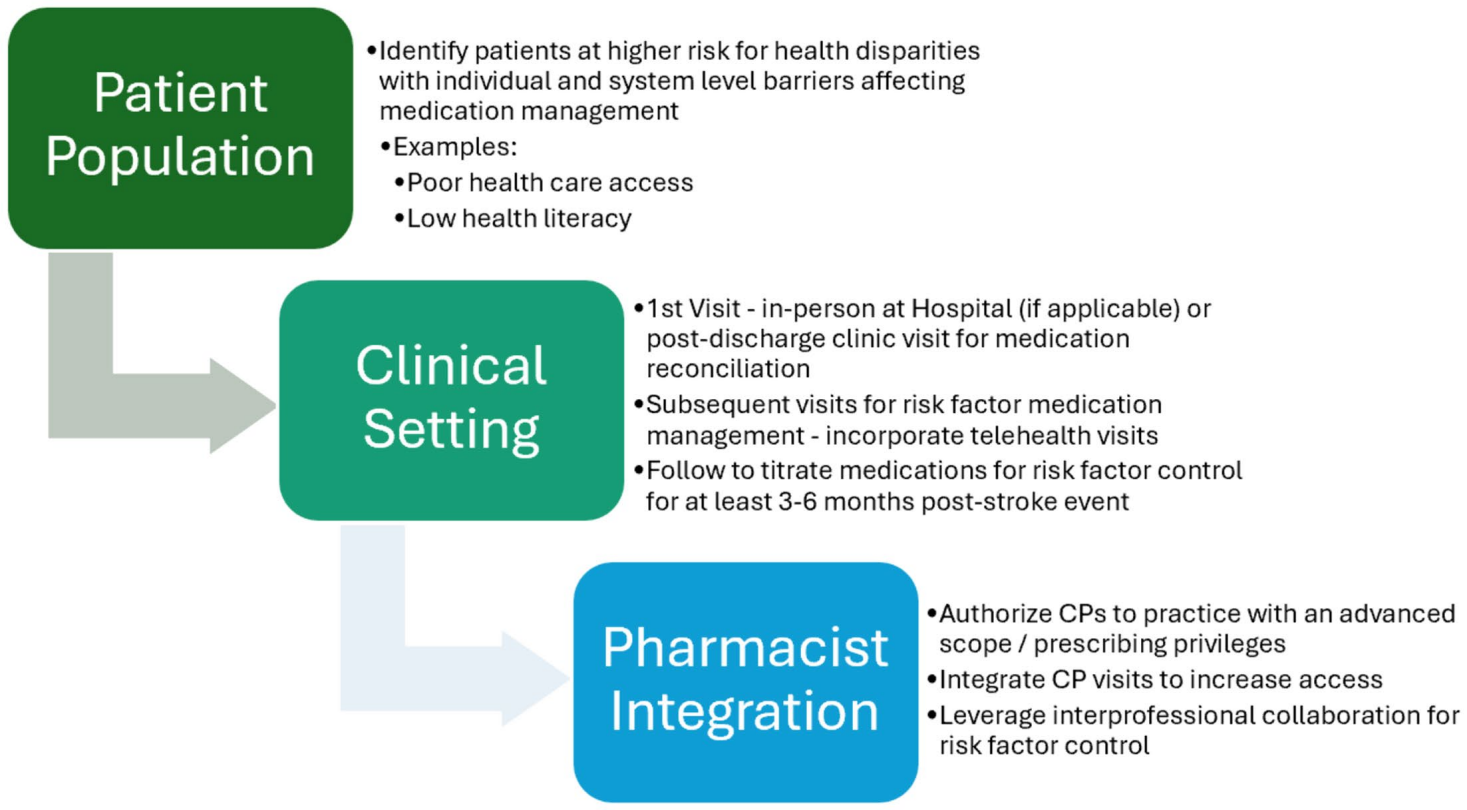
Proposed foundational elements for integration of clinical pharmacists within post-stroke ambulatory care teams.

Based on our findings from all selected trials, the study team established foundational elements that have been included in these trials to facilitate the integration of CPs within interprofessional post-stroke team models with the aim of enhancing patient care (Figure 4). The proposed framework recognizes the differences among systems and suggests flexibility when implementing the practice model. However, the core element remains the same: integrating CP services with an advanced scope to promote interdisciplinary co-management.

We found five completed RCTs (8, 21–24) and one RCT abstract that integrated CPs within outpatient secondary stroke prevention to provide value-based care (38) for patients (20). Of note, all trials enrolled fewer than 300 participants and none evaluated real-world effectiveness. Despite limited evidence, most trials showed improvements in clinical and patient-reported outcomes, particularly in surrogate markers (e.g., BP, glucose), and no studies reported harm from CP interventions.

Medication adherence outcomes were included in the majority of studies, but varied on if they were collected via medication fill history [e.g., Medication Possession Ratio (39)] or reported by the patient via questionnaires [e.g., Morisky Medication Adherence Scale (40)]. Future studies should consider combining both approaches for more robust adherence assessment.

CP integration has shown to be effective in managing chronic conditions, such as chronic kidney disease and mental health disorders (41–43). Of the studies included in this review, CPs primarily provided patient education, adherence support, and risk factor counseling. Few studies included CPs with prescribing authority, though such roles – often accredited through residency and/or board certification – have shown a positive impact on clinical and financial outcomes (44, 45).

Only four trials featured CPs with an advanced scope of practice, all in North America. One demonstrated a statistically significant improvement in BP and lipid control (23), while three are still pending results or are ongoing (26, 31, 32). Many countries may not have legislation to support collaborative agreements, which could affect the breadth of services that a CP can provide in team-based healthcare models globally. Regardless, this represents an opportunity to increase the utilization of CPs with appropriate training to take on expanded roles (41).

Three of the four trials that included CPs with prescribing authority also employed interdisciplinary models, suggesting these CP roles may complement team-based care. No trials included transdisciplinary models, where roles are shared across disciplines. While evidence is limited in stroke care, CPs have shown promise in transdisciplinary teams within ambulatory palliative care (46).

Telehealth has improved access to acute stroke management within healthcare systems, but inequities must be addressed in the delivery of ambulatory services (47). This mode of health service delivery has been shown to be equivalent or more clinically effective when compared to “usual care” across various disciplines and has been demonstrated to increase patient satisfaction with healthcare services (8, 48–50). Other studies have similarly demonstrated positive outcomes from incorporating CP services via telehealth for chronic disease management (51–53). The studies in this review also suggest that telehealth is a feasible mode of CP integration, with its use in over half (8 of 14 studies) (8, 20, 22, 24, 25, 28, 30, 31) of the included trials. Still, few studies assessed implementation feasibility or patient-centered outcomes, highlighting a gap in real-world applicability.

Barriers to CP integration may include regulatory limits, provider resistance, reimbursement issues, and lack of awareness of CP capabilities (54). Nonetheless, economic modeling from the 2015 RxACTION study, which assessed the impact of pharmacist-led antihypertensive medication management, showed that pharmacist interventions were associated with a cost savings of $1.137 trillion and could save an estimated 30.2 million patient life years over 30 years (45, 55). This study suggests that pharmacist-led care can produce significant cost savings and improve health outcomes, even if not specific to post-stroke care.

Most trials lacked data on patient race, ethnicity, socioeconomic status, or rural representation, underscoring the need for more inclusive research. Future analyses delineating CP contribution toward patient-oriented outcomes in multi-component interventions may help identify CP impact on reducing disparities and promoting healthcare equity in post-stroke secondary prevention.

## Strengths and limitations

Our team members from different disciplines collaboratively contributed to this body of research through scientific teamwork (56). The scoping review included multiple electronic databases and searched terminologies to be comprehensive. To maximize the utility of the review, all relevant clinical trial findings are reported, including ongoing trials reported in clinical trials databases. We can only speculate that there may be negative findings or lack of follow-up leading to data not being captured and reported.

Additionally, the included trials were heterogeneous, from how “usual care” was delivered in control groups, to categories of CP interventions and prescribing privileges, which precluded recommendations of a standardized approach. Differences in study design, such as the care system employed, frequency and time frame of interventions, and how outcomes were assessed may have caused discrepancies in findings, such as CP impact on medication adherence. Further, the lack of consistent reporting on healthcare disparities made it challenging to interpret the generalizability of reported outcomes. Our review’s definitions of urban, suburban, and rural were based on United States census information (34), which may have not been accurate for analyzing the setting of trials in other countries.

Finally, some assumptions were made in reporting CP interventions in each trial, as most did not report CP services in specific detail. Therefore, we may not have captured all services performed by CPs. Future trials should quantify specific CP interventions contributing specifically to overall study outcomes.

## Conclusion

Our review of the available evidence demonstrates that the addition of CPs may lead to improved clinical and patient-centered outcomes in secondary stroke prevention, but studies with fully reported results are limited. Team-based models have the potential to provide value-based care and optimize healthcare systems. It is evident that ambulatory CPs are being effectively integrated into different collaborative team-based models within these globally conducted trials. Herein lies an opportunity for purposeful utilization of CP services to reduce health inequities in post-stroke care and assess their impact in real-world settings. This should be informed by adequate trial reporting of study outcomes among minoritized populations to inform equitable health care policy.

## Data Availability

The original contributions presented in the study are included in the article/supplementary material, further inquiries can be directed to the corresponding author.
